# Cytomolecular characterization of *de novo* formed rye B chromosome variants

**DOI:** 10.1186/1755-8166-5-34

**Published:** 2012-07-16

**Authors:** André Marques, Sonja Klemme, Marcelo Guerra, Andreas Houben

**Affiliations:** 1 Leibniz-Institute of Plant Genetics & Crop Plant Research (IPK), 06466, Gatersleben, Germany; 2 Laboratory of Plant Cytogenetics and Molecular Biology, Department of Botany, UFPE, Recife, Brazil

**Keywords:** Supernumerary chromosome, Rye, B chromosome polymorphisms

## Abstract

**Background:**

B chromosomes (Bs) are dispensable elements which occur in many species including rye (*Secale cereale*). We determined the organization of B variants to obtain insights into the origin of B polymorphisms in rye.

**Results:**

The observed B variants were classified according to their morphology and *in situ* hybridization patterns with the B-specific repeats D1100 and CL11 into (I) long arm iso B, (II) D1100-deficient B and (III) small metacentric B variants. Long arm iso Bs are likely products of a meiotic centromere misdivision and subsequent duplication of the long arm, whereas small B variants are probably generated by chromosome breakage. Some deficient Bs experienced extensive amplification of CL11 repeats.

**Conclusions:**

Both the pericentromere and the nondisjunction control region seem to be involved in the generation of rye B chromosome variants. However, due to the loss of the B-specific nondisjuction control region most of the variants generated are not capable to accumulate in a population.

## Background

B chromosomes (Bs) are dispensable elements and occur in many species of plants, fungi and animals over a wide geographical distribution [1]. Although Bs have intensely been cytologically investigated since their discovery a century ago [2], little is known about their origin, mode of evolution and molecular composition. According to most views, Bs are selfish elements which have arisen from normal A chromosomes (As) and maintain themselves through generations by accumulation mechanisms [3].

Due to their mainly neutral situation in host genomes it is expected to observe B chromosome polymorphisms among populations. The origin of B structural variants is mostly attributed to be of monophyletic origin from a unique type of ancestral B chromosome which afterwards diverged in different types through generations [4,5]. Indeed, there are several cases of B polymorphisms whether numerical or structural [6,7]. For the B chromosome of the grasshopper *E. plorans* a large variety of structural variants has been demonstrated among many populations [6]. In many plants, B polymorphisms have been only attributed to numerical polymorphisms [7,8]. Although, in a few cases B structural variants in natural populations have been identified e.g. *Brachycome dichromosomatica*[4] and *Scilla autumnalis*[9].

Rye is an excellent system for B polymorphism studies, because of its wide distribution across Europe, Middle East and Asia [10]. The standard B is acrocentric and smaller than A chromosomes of rye. Apart of the terminal region of the long B arm, a high level of overall similarity exists between As and Bs of rye [11,12]. Two rye B-specific high copy repeats (D3900 and E1100) are clustered on its terminal region of the long arm [13,14]. More recently, a B-pericentromeric repeat CL11 has been identified (Martis et al., unpublished).

Naturally occurring B variants may be produced from the standard type by misdivision of the centromere or by deletions of segments from the long arm of the B [15,16] generating mainly isochromosomes, both of the short arm and of the long arm B, as well as deficient Bs [17]. Often due to the loss of the terminal end of the long B arm directed nondisjunction is impaired and therefore these B variants are not able to accumulate [16,18]. Hence, structural variants are rare in natural populations, indicating that the standard form is the only one maintained in the long term [19]. We have found with high frequency in the progeny of one plant of the rye line 7415 [20] structural rearrangements of the Bs. In order to obtain insights into the origin of the rye B polymorphisms we characterized the various B variants in relation to the two rye B-specific high copy repeats (CL11 and D1100).

## Results and discussion

**Figure 1:**
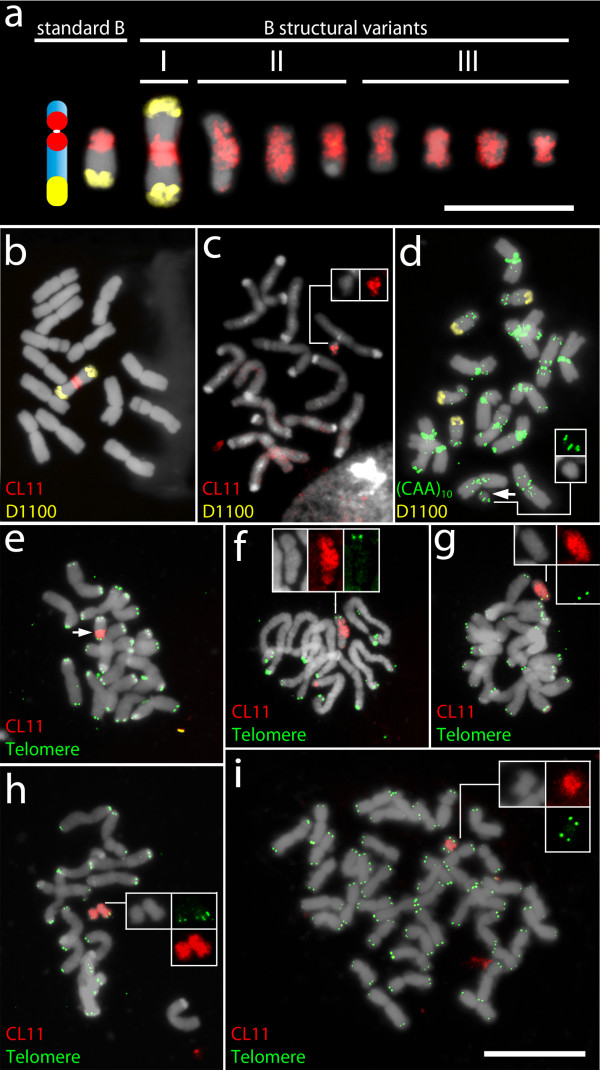
**Characterization of mitotic rye (*Secale cereale*) B chromosome variants by FISH with the B-specific probes D1100 and CL11, the microsatellite (CAA)**_**10**_**, as well as with the *Arabidopsis*-type telomere repeat.** (**a**) Standard Bs and its structural variants found in the rye line 7415. The B variants are classified into three different subtypes. Bar equals 10 μm. (**b**) Plant #11 carrying a long arm iso B, (**c**) Plant #4 carrying a small deficient B lacking the long arm, (**d**) Plant #6 carrying four standard Bs and one small B variant (arrow). (**e**) Standard B showing telomeric signals on both arms and a pericentromeric CL11 signal, (**f**) B-variant almost entirely labeled with CL11 sequence but with telomeric signals on one chromosome arm only, (**g**) B variant showing a high accumulation of CL11 signals and telomeric signals only on the short arm, (**h**) two small B variants, (**i**) metaphase of a wheat plant with rye short arm iso B showing telomeric signals on both arms of a small iso B. Inserts highlight B variants. Bar equals 20 μm.

The analysis of 16 offsprings derived from one selfed +2B plant revealed eight seedlings carrying B chromosomes. Three out of these eight plants presented 1 up to 4 standard Bs whereas the other five plants exhibited extra-chromosomes differing in size and morphology from the standard Bs. Three plants showed a mosaic of different types of extra-chromosomes. Cross-hybridization with the B-specific probes CL11 and D1100 revealed the B-origin of the extra chromosomes, which were classified according to their morphology and hybridization patterns into (I) long arm iso B, (II) D1100-deficient B and (III) small metacentric B variants (Figure 1a). Table 1 shows the intraindividual variation of B structural rearrangements found in five individuals of rye.

**Table 1:** Number and types of structural B variants found in five individuals of the rye line 7415

**Individuals**	**N° of Bs per cell**	**Identified B variants**				**Number of cells analyzed**
		**Standard B**	**Small metacentric B**	**Large iso B**	**Deficient B**	
**7415/1**	1	0	0	23	0	23
**7415/4**	1	0	11	0	10	21
**7415/5**	1–2	2	10	0	12	24
**7415/6**	3–4	20	8	0	0	28
**7415/11**	1	0	0	27	0	27

We found the standard Bs to be mitotically unstable in two plants of the progeny of one plant from the line 7415, undergoing different levels of chromosome deficiency. The morphology and number varied even between cells of the same plant. As a result we found fragmented B chromosomes with and without additional standard Bs per cell (Table 1, individuals #5 and #6). This is unusual as the standard B chromosome of rye is generally mitotically stable. However, the isochromosomes of the long arm were mitotically stable, since no additional fragmented B chromosomes have been found in those plants (Table 1, individuals #1 and #11).

The high frequency (approx. 63%) of B structural rearrangements in this progeny is unusual. However, structural rearrangements of rye Bs have been reported after experimental crosses [21]. Two out of five individuals carried a long arm iso B with both terminal regions labeled with D1100 signal (Figure 1b, type I). The origin of the observed long arm iso Bs is most likely related to meiotic centromere misdivision due to formation of univalent Bs. Half of the small B variants appeared to be short arm telosomes or chromosomes with a partial loss of the long arm (Figure 1f, g; type II). The other B variants revealed a metacentric morphology (Figure 1d, h; type III). In order to confirm the isochromosomal nature of the small metacentric B variants, the B-short arm marker (CAA)_10_ was used for FISH. Microsatellite-specific signals were found on the A chromosomes, mainly located on their proximal regions and on the short arm of the standard B. Surprisingly, only one arm of the small metacentric B variants showed (CAA)_10_ signals (Figure 1d), suggesting that these chromosomes are not short arm iso Bs, but rather chromosomes with a partial loss of the long arm. In rare cases, deficient Bs were almost entirely labeled with CL11-specific signals, but were found lacking the nondisjunction control region, (Figure 1f, type II). The extended CL11 signal suggests an extensive amplification of this sequence beyond the pericentromere. Generally DNA amplification occurs during periods of genomic instability and several amplification mechanisms have been suggested [22].

Next, we tested whether the truncated B arms are stabilized by *Arabidopsis*-type telomere repeats. As positive control we employed the wheat-rye addition line B^s-2^ containing short arm B isochromosomes (Figure 1i). Standard Bs and the iso-short arm B of line B^s-2^ are characterized by *Arabidopsis*-type telomeric signals on the termini of both arms (Figure 1e). All small, newly formed B variants displayed telomeric signals only in one arm (Figure 1f–h), confirming that the small Bs are results of chromosome breakage events and that no telomere repeats or only few are sealing the broken arm.

Both the pericentromere and the nondisjunction control region seem to be involved in the generation of B variants. However, most of the variants generated are not capable to accumulate in a population. The only B variant which maintains its nondisjunction capacity is the long arm iso B type [23]. However, the maintenance of large iso Bs has not been found in natural populations, perhaps because isochromosomes frequently show centromere dysfunction and/or meiotic irregularities with iso-ring formation at metaphase I [6,24]. Hence, most B variants of rye will undergo elimination within the population despite the mainly neutral effect of Bs on the host genome.

## Material and methods

### Plant material

The self-fertile *Secale cereale* inbred line 7415 carrying B chromosomes of the Japanese JNK strain were analyzed [20]. The *T. aestivum* L. (‘Chinese Spring’) addition line B^s-2^ containing short arm B isochromosomes was used as control [23]. Seeds were germinated on humid filter paper.

### Probe preparation and fluorescence in situ hybridization (FISH)

The B-specific high copy repeats D1100 [14] and CL11 (Martis et al., unpublished), located on the terminal nondisjunction control region and on the pericentromeric region, respectively, were amplified by PCR and the products were cleaned using a QIAquick PCR Purification Kit (Qiagen). The telomeric probe was obtained from a plasmid containing an *Arabidopsis*-like telomere TTTAGGG sequence. All probes were labeled with Texas red-dUTP (Perkin Elmer) or Alexa-488-dUTP (Invitrogen) directly by nick translation. Additionally, a (CAA)_10_ oligonucleotide directed labeled with Cy3 (MWG, Eurofins) was also used as FISH probe. Slide preparation and *in situ* hybridization were performed as described [25]. Microscopic images were recorded using an epifluorescence Leica DMLB microscope equipped with a Cohu CCD camera or alternatively an Olympus BX61 microscope equipped with an ORCA-ER CCD camera. Images were analyzed using the QFISH software (Leica) or SIS software (Olympus), respectively. The monochromatic images were pseudocolored and merged using Adobe Photoshop CS5.

## Abbreviations

FISH: Fluorescence *in situ* Hybridization; DAPI: 4′,6-diamidino-2-phenylindole.

## Competing interests

The authors declare that they have no competing interests.

## Authors’ contributions

AM carried out studies. SK participated in the design of the study. MG and AH conceived of the study, and participated in its design and coordination and helped to draft the manuscript. All authors read and approved the final manuscript.
